# The development and initial feasibility testing of D-HOMES: a behavioral activation-based intervention for diabetes medication adherence and psychological wellness among people experiencing homelessness

**DOI:** 10.3389/fpsyg.2023.1225777

**Published:** 2023-09-19

**Authors:** Katherine Diaz Vickery, Becky R. Ford, Lillian Gelberg, Zobeida Bonilla, Ella Strother, Susan Gust, Edward Adair, Victor M. Montori, Mark Linzer, Michael D. Evans, John Connett, Michele Heisler, Patrick J. O'Connor, Andrew M. Busch

**Affiliations:** ^1^The Health, Homelessness, and Criminal Justice Lab, Hennepin Healthcare Research Institute, Minneapolis, MN, United States; ^2^Department of Medicine, Hennepin Healthcare, Minneapolis, MN, United States; ^3^The Quorum for Community Engaged Wellness Research, Minneapolis, MN, United States; ^4^Department of Family Medicine, David Geffen School of Medicine at UCLA and UCLA Fielding School of Public Health, Los Angeles, CA, United States; ^5^School of Public Health, University of Minnesota, Minneapolis, MN, United States; ^6^Division of Endocrinology, Diabetes, Metabolism, Nutrition, Department of Internal Medicine and the Knowledge and Evaluation Research Unit, Mayo Clinic, Rochester, MN, United States; ^7^Clinical and Translational Science Institute, University of Minnesota, Minneapolis, MN, United States; ^8^Division of General Medicine, Department of Internal Medicine, University of Michigan, Ann Arbor, MI, United States; ^9^Center for Chronic Care Innovation, HealthPartners Institute, Bloomington, MN, United States; ^10^The Behavioral Health Equity Research Group, Hennepin Healthcare Research Institute, Minneapolis, MN, United States

**Keywords:** diabetes, health equity, homelessness, behavioral trials, behavioral activation trauma, behavioral activation

## Abstract

**Introduction:**

Compared to stably housed peers, people experiencing homelessness (PEH) have lower rates of ideal glycemic control, and experience premature morbidity and mortality. High rates of behavioral health comorbidities and trauma add to access barriers driving poor outcomes. Limited evidence guides behavioral approaches to support the needs of PEH with diabetes. Lay coaching models can improve care for low-resource populations with diabetes, yet we found no evidence of programs specifically tailored to the needs of PEH.

**Methods:**

We used a multistep, iterative process following the ORBIT model to develop the Diabetes Homeless Medication Support (D-HOMES) program, a new lifestyle intervention for PEH with type 2 diabetes. We built a community-engaged research team who participated in all of the following steps of treatment development: (1) initial treatment conceptualization drawing from evidence-based programs, (2) qualitative interviews with affected people and multi-disciplinary housing and healthcare providers, and (3) an open trial of D-HOMES to evaluate acceptability (Client Satisfaction Questionnaire, exit interview) and treatment engagement (completion rate of up to 10 offered coaching sessions).

**Results:**

In step (1), the D-HOMES treatment manual drew from existing behavioral activation and lay health coach programs for diabetes as well as clinical resources from Health Care for the Homeless. Step (2) qualitative interviews (*n* = 26 patients, *n* = 21 providers) shaped counseling approaches, language and choices regarding interventionists, tools, and resources. PTSD symptoms were reported in 69% of patients. Step (3) trial participants (*N* = 10) overall found the program acceptable, however, we saw better program satisfaction and treatment engagement among more stably housed people. We developed adapted treatment materials for the target population and refined recruitment/retention strategies and trial procedures sensitive to prevalent discrimination and racism to better retain people of color and those with less stable housing.

**Discussion:**

The research team has used these findings to inform an NIH-funded randomized control pilot trial. We found synergy between community-engaged research and the ORBIT model of behavioral treatment development to develop a new intervention designed for PEH with type 2 diabetes and address health equity gaps in people who have experienced trauma. We conclude that more work and different approaches are needed to address the needs of participants with the least stable housing.

## 1. Introduction

The devastating impacts of homelessness and housing instability on diabetes management, outcomes, and mortality have become increasingly clear. While the prevalence of diabetes among people who have experienced homelessness (PEH) appears similar to that of the general population (Bernstein et al., 2015), PEH with diabetes experience lower rates of ideal glycemic control (Axon et al., 2016) and are hospitalized for diabetes complications an average of 10 years sooner than their housed peers (Adams et al., 2007). This results in premature death among PEH with 3- to 6-fold higher death rates due to diabetes (Baggett et al., 2013; Health Homelessness Criminal Justice Lab, 2023). We recently detailed the drivers of these disparities with qualitative data and found that the combined impact of individual and structural barriers to diabetes self-care and overall wellness were rooted in a lack of autonomy and security, unpredictable routines, and lack of supportive relationships and accessible resources (Turcotte Manser et al., 2023).

Behavioral health comorbidities present further challenges to diabetes management for PEH. Our team previously documented a 106% increase over 19 years in the overlap of physical, mental, and substance use conditions among PEH in Minnesota (Vickery et al., 2021). Trauma is notably present in the lives of PEH. A detailed national survey of 540 PEH across 5 U.S. cities found that 62% of respondents had witnessed a violent attack and 49% had experienced a violent attack (Meinbresse et al., 2014; Robinson, 2014). Furthermore, interdisciplinary scholarship increasingly documents homelessness itself as a source of trauma specifically via the psychological distress of losing one's home, the conditions of life in many shelters, and the association of loss of housing with physical and sexual abuse (Goodman et al., n.d.). Scholars continue to explore the role of trauma and abuse in increasing lifetime risk for obesity (Lindert et al., 2014) and diabetes (Thomas et al., 2008, p. 2; Rich-Edwards et al., 2010).

The American Diabetes Association (ADA) currently recommends, with the highest level of evidence, that diabetes providers assess housing insecurity/homelessness “to inform treatment decisions” (American Diabetes Association Professional Practice Committee, 2021). Yet, we found limited evidence to inform how diabetes treatment decisions should be adapted to meet the needs of PEH. One qualitative study of experienced providers recommended the use of support workers and outreach into shelters (Campbell et al., 2020). Another qualitative study with patients used community-engaged approaches to document the combined demands of homelessness and diabetes on patients' psychological wellness and mental health which impaired their ability to focus on diabetes self-management (Campbell et al., 2021). This research is limited in scope, and we found no randomized clinical trials of interventions targeted to the needs of PEH living with diabetes.

The ADA specifically recognizes “lay health coaches,” and recommends patients experiencing homelessness be provided this support “when available”, however, they do not define this term. Barnett et al. (2018) describe lay health workers, a widely accepted synonym for coaches, as “interventionists without formalized…training who generally are from the same community as the population they serve.” Lay health coaches frequently combine health education with psychosocial support and behavior modification techniques to meet client-determined goals (McQueen et al., 2020). While many diabetes coaching and support interventions have demonstrated effectiveness for people in low-resource communities (Shah et al., 2013), people experiencing homelessness are rarely included in such longitudinal trials due to the unpredictable nature of their lives, lack of support for communication, and other specific requirements of research protocols. We found no diabetes coaching programs that specifically addressed the social risk of homelessness and its associated trauma. Given such challenges to engagement in behavior change and longitudinal research, we adopted a community-engaged research approach to design and test a diabetes coaching intervention to support lifestyle and pharmacologic management of diabetes in PEH.

In this study, we detail our efforts to develop an evidence-based coaching intervention tailored to the needs of PEH living with type 2 diabetes using the ORBIT model of behavioral treatment development (Czajkowski et al., 2015). Given the breadth of behaviors involved in diabetes self-care (Shubrook et al., 2018), the difficulties noted by our team in achieving diet/exercise changes while experiencing homelessness, and the known impact of low-cost diabetes medications, we decided to focus on medication adherence as the intervention target. Any behavior related to getting and taking medications as prescribed was included as treatment targets when appropriate for the individual patient (e.g., pill taking, insulin or GLP-1RA injections, proper medication storage, blood sugar checks, seeking prescriptions/refills, etc.). We named our program **D**iabetes **Ho**meless **Me**dication **S**upport (D-HOMES). In this study, the multiple steps of intervention development we have undertaken have been detailed, all guided by a community-engaged research team including (1) conceptualization, (2) formative qualitative research, and (3) pilot testing in an open (single-arm) trial (Table 1). We received human subjects' approval for all stages of this study before beginning any activities from the Hennepin Healthcare Research Institute Institutional Review Board.

**Table 1:** Intervention development steps.

**Community-engaged research team**	**Step of intervention development**	**Description of study steps**	**ORBIT model phase**
Ongoing, iterative feedback from the consistent team with lived experience	Step 1: Conceptualization	Initial manual development	1a
	Step 2: Formative intervention development	Qualitative interviews with target patients and providers	1b
	Step 3: Pilot test	Manual demonstration in an open trial with post-intervention qualitative feedback	2

## 2. Methods + results of treatment development steps

### 2.1. Community-engaged research team

Aligned with the values of community-based participatory research (Israel et al., 2010), we convened a team of people with various experiences of type 2 diabetes and housing instability as a first step in this research inquiry. The team named itself “Quorum,” and participants of the initial Quorum team included Medicaid recipients who had experienced type 2 diabetes and homelessness, a facilitator with expertise in community-based participatory research, as well as community health workers from our health system. Facilitation involves substantial time to build trust between researchers and community members on the team, bidirectional learning, and established decision-making processes that all team members agree to follow. Before we obtained funding (K23DK118117), this team helped to identify the direction of this work (i.e., lay health coaching intervention focused on PEH with type 2 diabetes). Once funded, the Quorum team grew to include more representation from people living with diabetes who had experienced homelessness and housing and healthcare service providers with relevant experience. The team has met monthly since 2016 and has had direct input on all steps of the intervention development process influenced by the collaborative intervention planning framework (Cabassa et al., 2011, 2014). Community members were paid for their participation in all meetings and workshops, offered meals, and invited as guests to conferences and webinars to share information about the treatment development process. Congruent with the ethics of community-engaged research, this team served as key advisors to the research but were not research subjects, and, therefore, this work was not subject to IRB approval (Khodyakov et al., 2016). The team's impact on each step of our treatment development process will be summarized within each sub-section below and summarized in Table 2.

**Table 2:** Summary of intervention development decisions and supporting data.

**Intervention development decision**	**Source of data informing decision**			
	**Community-engaged research team**	**Patient interviews**	**Provider interviews**	**Open trial results**
Targeting primarily medication adherence		X	X	X
Use of behavioral activation	X	X	X	
Integrated mood/behavior approach	X	X	X	X
Interventionists called “Diabetes wellness coach” (not case managers and counselors)	X	X	X	
Flexibility in intervention logistics (location, modality, and number of sessions)	X	X		X
Personalized “hand-offs” for recruitment	X			X
Increased recruitment of people in transitional/supportive housing		X	X	X
Creation of a recruitment video featuring team members of color	X			X
Simplified consent documents with consent quiz	X			X
Choice of simple educational materials with engaging graphics, video options	X	X		X

### 2.2. Step 1: conceptualization

The team developed criteria to follow when initially conceptualizing D-HOMES:

- The need to support the overlap of physical and behavioral health conditions (“the pile up” in the words of the Quorum team): Prior work by our team (Vickery et al., 2021) and others (Baggett et al., 2013; Fazel et al., 2014) has demonstrated the substantial multi-morbidity experienced by PEH especially the overlap of physical, mental health, and substance use conditions. This is especially important when considering the known bi-directional relationship between medication adherence and behavioral health (Kushel, 2023). Therefore, we looked for models aligned with our treatment priorities that use health coaches to support health behavior change in the context of concurrent medical and behavioral health concerns.
- A model that would offer support, not add stress, to the lives of PEH. We committed to finding a model that simultaneously targeted psychological wellness and chronic disease self-management. Recommended diabetes self-care activities can take up to 4 h per day (Shubrook et al., 2018)—a lot of time for anyone to take on. When added to the stress of housing instability, our team emphasized the need to ensure D-HOMES supported people's psychological wellness overall and did not just push improved diabetes care at the expense of other healthcare needs. This also aligned with a scientific need for a transdiagnostic concept given the variety of psychopathology sometimes lacking a formal diagnosis.
- Coaches who could build trusted relationships with participants with the skills and knowledge to navigate existing support programs and housing services. This was informed by housing outreach staff, a nurse from Health Care for the Homeless, and community health workers on our team. This led us to build an intervention that can be delivered by those with the level of training common for staff working in supportive housing settings, i.e., non-mental health or heathcare experts.
- Alignment with the philosophy of harm reduction used in the U.S. Health Care for the Homeless Program. Developed with populations who use substances, harm reduction takes an approach that “meets people where they're at.” In other words, supporting people's cultural identities and encouraging them to take whatever next steps they feel will benefit them (within reason) without coercion from healthcare professionals or others.

We determined that behavioral activation (BA) appeared to be a good fit for an intervention approach addressing diabetes medication adherence and psychological wellness among PEH. Originally designed as a psychotherapy to treat major depression, BA seeks to overcome avoidant or isolating patterns with structured engagement in pleasurable and valued activities (Kanter et al., 2009, 2010). Strong evidence supports its ability to benefit psychological wellness (Mazzucchelli et al., 2010). However, more recently, BA has been applied to a broad array of populations and behavior change targets. Most relevant to the current investigation, BA has been successfully applied to target health behavior change (e.g., smoking cessation, substance use recovery, exercise engagement, etc.; Busch et al., 2017; Ciccolo et al., 2022; Adkins-Hempel et al., 2023) in various populations with high rates of behavioral comorbidities, psychosocial distress, and psychosocial barriers to change [e.g., those in residential substance abuse treatment, patients with HIV (Pinkston et al., 2022), and low income urban Black men (Ciccolo et al., 2022)]. This literature includes evidence that BA can improve medication adherence among people living with HIV and substance use (Daughters et al., 2010) and in other resource-limited settings (Magidson et al., 2019) as well as to support diabetes self-management specifically (Egede et al., 2021). Bachelor's level (or equivalent) providers with appropriate training can feasibly deliver BA with high fidelity (Chowdhary et al., 2016). Furthermore, as we reviewed materials and assembled the treatment manual, we found synergy in language and approach between BA's focus on personal values and graded goal setting and healthcare for the homeless' patient-centered and harm reduction philosophy.

**Figure 1:**
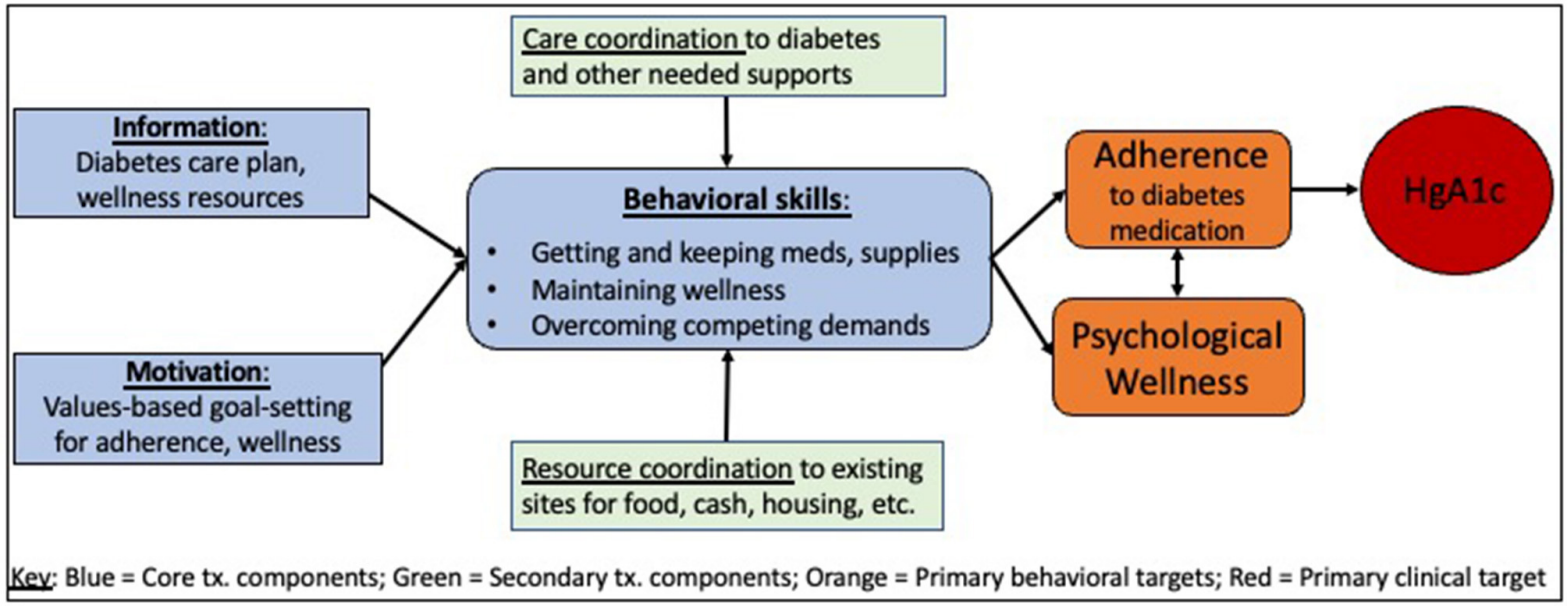
Diabetes Homeless Medication Support (D-HOMES) treatment model.

We used the Information-Motivation-Behavioral (IMB) skills model to guide our intervention design. The IMB outlines the behavior of medication adherence as a product of the combined information and motivation that leads to the behavioral skills needed to adhere (Fisher et al., 2009; Nelson et al., 2018). Our treatment manual prompts coaches to provide specific support to ensure each participant has access to appropriate *information* about their diabetes care plan and any needed wellness resources. Coaches also assess each participant's *motivation* to improve medication adherence and explore values to ensure their work remains aligned with the participants' source of meaning in their lives. *Behavioral skills* are noted as coaches conduct initial sessions with participants, and tailored support is provided to improve any missing skills. We situated the IMB model within the socio-ecological framework to better characterize the multi-level factors that impact an individual's medication adherence and diabetes self-management (Fisher et al., 2005; Hill-Briggs et al., 2021). Importantly, our study took place in a U.S. urban area with widely available health insurance (Medicaid), access to primary care and behavioral health services (including integrated, walk-in care at a local Health Care for the Homeless program clinic site), affordable and accessible pharmacies, and supportive housing options (shelters, transitional, and supportive housing facilities). Furthermore, we consulted conceptual models outlining competing demands as key barriers to chronic disease management and healthcare access for people in low socioeconomic groups (Gelberg et al., 1997, 2000; Shippee et al., 2012). We illustrate our intervention treatment model and targeted outcomes in Figure 1.

We planned that our target population for the D-HOMES program would be people experiencing all types of housing instability. We use the federal (HEARTH Act) definition which broadly defines homelessness as encompassing the spectrum of housing instability which includes, for example, worry about the inability to pay rent or staying in single and scattered site transitional or permanent supportive housing, emergency shelters, outdoors, or in other places not meant for human habitation (HUD Exchange, 2011).

We began with the medication adherence module of an existing BA treatment manual, Behavioral Activation for Health and Depression (BA-HD; Gathright et al., 2022). We integrated into the manual descriptions of the Health Care for the Homeless care model (Zlotnick et al., 2013), harm reduction philosophy (Meges et al., 2014), and pulled from the National Health Care for the Homeless Council's clinical guidance on diabetes management (Richert et al., 2019). We added a resource guide to direct coaches to support participants to access affordable medication, free/low-cost meals, groceries, and other material goods, and walk-in physical and behavioral healthcare at the local Health Care for the Homeless and other locations. Based on the existing BA manual, we developed an initial plan to offer 10 sessions over 12 weeks delivered by a one-on-one interventionist. Sessions could be in-person, by phone, or by video-based delivery. We planned to offer one commercially available tool to support medication adherence goals (e.g., pillbox with morning, mid-day, and evening boxes for a patient on 3x/day medications), but we explored which tools participants might desire in the formative qualitative research step detailed below.

#### 2.2.1. Community-engaged research team impact

During Step 1, the Quorum team reviewed the treatment model and planned qualitative approaches. They emphasized the sensitive nature of diabetes and its connection to shame, grief, and painful memories in their lives. They advised us to use recruitment methods emphasizing “warm handoffs” with a direct connection to housing, healthcare, or other staff and research study team members as much as possible. The team also reinforced the plans for flexible attempts to connect with people in-person, by phone, or video. The team engaged with coaches and study staff to prepare them to work with the target population. After incorporating their feedback, the conceptual model and initial intervention plan had face-value validity with our community-engaged research team.

We planned Steps 2 and 3 to gather qualitative and quantitative evidence of this program's feasibility and acceptability with PEH with type 2 diabetes. Steps 2 (formative qualitative research) and 3 (pilot testing) were conducted only after approval by the Hennepin Healthcare Research Institute's Institutional Review Board.

### 2.3. Step 2: formative qualitative research

We designed one-time cross-sectional qualitative interviews and focus groups to inform the adaptation of our planned manual to the needs of people living with type 2 diabetes who had experienced homelessness (Rosen et al., 2017).

#### 2.3.1. Focus groups with patient participants

##### 2.3.1.1. Methods—data collection

Participants who responded to posted flyers, individual invitations by housing and healthcare providers, and recruitment letters had follow-up screening phone calls. Participants were adults who met the following inclusion criteria: (1) self-reported type 2 diabetes diagnosis by a medical provider, (2) hemoglobin A1c testing at a certified medical laboratory in the past 90 days and/or point-of-care testing at recruitment without an eligibility cutoff, (3) self-report of homelessness in the last 12 months (per the Hearth Act; HUD Exchange, 2011), positive homeless address indicator in electronic health record (Vickery et al., 2017), or encounter at a Health Care for the Homeless clinic in the past 12 months, and (4) English language conversational fluency. Exclusion criteria included inability to consent due to (1) active intoxication, (2) active psychosis or dementia, or (3) active legal commitment. Interviews were conducted by a facilitator trained in qualitative research methods (KDV, LJ) between August 2019 and February 2020 at locations convenient to participants (e.g., homeless drop-in centers, shelters, and the public library) following a structured interview guide. We used a modified eco-map poster (see Supplementary material) to prompt discussion about “people, places and things” that represent participants' experiences with diabetes and the classification of these items as to whether they made it easier or harder to care for their diabetes (Hartman, 2003). We asked for specific participant feedback about the proposed intervention. More details about participant interview methods (including a detailed description of topics/questions) and results are available elsewhere including identified barriers and facilitators to diabetes medication adherence and self-care (Turcotte Manser et al., 2023). We offered transportation support (parking voucher or taxi), a stipend ($30 gift card), and a meal or snack to all participants.

Interviewers audio-recorded all sessions and noted their observations and impressions during data collection. Structured questionnaires recorded interviewees' demographic characteristics and assessed medication burden, comorbidities, and psychological distress using measures under consideration for use in the planned trial. Domains of interest for all interviewees included the use of BA, number, duration, and format (in-person, phone, or virtual) of intervention sessions, what to call the interventionist, and potential health behavior change goals. Interviewees were specifically asked about their preferred learning styles and desired tools to support health behavior.

##### 2.3.1.2. Methods—data analysis

Audio recordings were professionally transcribed verbatim and reviewed by interviewers for accuracy. Memos were collected and reviewed by the PI and mentors (AB and ZB). We summarized questionnaire data using descriptive statistics in Excel and R (R: The R Project for Statistical Computing, n.d.). We conducted a formal content analysis using a deductive, modified framework matrix analysis approach which is a method that includes (1) familiarization with the interviews by reading full transcripts and memos, (2) top coding of transcripts according to pre-defined domains representing themes of importance (in this case based on the interview guide), (3) summary of content and organization of codes into cells aligned with interview guide domains, and (4) summary of domains across all interviews (Gale et al., 2013). This efficient, effective technique summarizes the findings of qualitative data and is appropriately used in structured qualitative inquiries with clearly identified outcomes of interest such as treatment development (Smith and Firth, 2011). Summaries of each domain allowed for the rapid translation of qualitative data into adaptions to the D-HOMES intervention.

##### 2.3.1.3. Results

We conducted focus groups or qualitative individual interviews with participants (*N* = 26, 5 groups each with 3–6 participants and 2 individual interviews) from August 2019 to February 2020. Participants had a mean age of 55 years (range 39–74) and 42% identified as female. The majority identified as Black race (62%). Participants took a mean of 8 separate medications (range 2–20). Thirteen of 26 participants (50%) took oral medications and insulin, and another 9 participants (35%) took only oral medications for diabetes. A majority of participants reported one or more co-morbid mental illnesses diagnosed by a medical provider (Depression 69%, Anxiety Disorder/Panic 54%, PTSD 31%, Bipolar Disorder 19%; and Schizophrenia/Schizoaffective disorder 8%). Approximately 69% of participants met the screening cutoff for PTSD symptoms using the Brief Trauma Questionnaire (Supplementary Table 1; Schnurr et al., 1999).

We identified the following key conclusions from the patient focus group and interview data:

1. The need for integrated mood and health behavior management with frequent, bidirectional interactions between psychological wellness (primarily mood) and diabetes self-management in general and specifically with regard to diabetes medication adherence. Participants detailed that changes in their blood sugar impacted their moods as well as that anger, stress, and depression made it harder to take their medications and engage in other diabetes self-care activities. One participant told us “*Well, a lot of times too, when you get stressed out you also get depressed. And when I get depressed, I don't take my medication. I don't do anything.”* One described, “*They gave me [insulin] pens; I would throw them in the garbage can because I don't need it…I just gave up on everything.”* Another participant detailed her need to “*remind myself every day, like, “Okay, it's self-care.” You know, “Do your meds and do your…wash your face.” You know what I mean? You feel better when you do those things.”* Several participants described suicidal ideation as directly contributing to non-adherence as a form of passive suicidal behavior. One participant told us, “*I didn't take any of my diabetes medicines for like six years because I wanted to kill myself.”*
2. Patient participants emphasized the need to be ready to support people in the interaction of diabetes with substance use, real or perceived, and the associated stigma. Several participants noted that having or using insulin needles led to a presumption by housing and healthcare providers that they were using intravenous drugs. Several people additionally noted that when they used substances, their diabetes self-care and adherence suffered. As one explained, “*Basically, when you're doing that type of activity you don't – you're not worried about your health. Or you're not worried about taking your meds*.”
3. Patient participants clearly preferred to avoid traditional mental health labels for the interventionist such as “counselor” or “psychologist” given the stigma against and misunderstandings of talk therapy. As one participant explained, “*I don't want no…psychologist where the only thing I'm doing is talking.”*
4. Patient participants noted the instability of shelters posing substantial barriers to diabetes self-care and adherence and some suggested timing the intervention with the receipt of housing. One participant told us, “*I mean, I try to do it [diabetes self-care including medication adherence] even [when] homeless, but it's harder doing it. But having your own place, you've been more stable. Yes, it helps a lot. Especially if you're serious about getting your health together.”* Although, another participant in the same focus group wanted help even before housing, noting her diabetes worsening added to her stress and fear while staying in her car.
5. Patient participants had divided opinions on whether the intervention should focus on medication adherence alone vs. all diabetes self-care. One told us, “*It's hard in the street for diet and exercise. So, I would say maybe they could support you with the medicine.”* Yet, the majority of patients felt that offering support for medication adherence had to be accompanied by attention to support people in healthy eating, physical activity, and psychological wellness.

Further findings were also reinforced by providers, see *Results* below.

#### 2.3.2. Individual interviews with housing and healthcare providers

##### 2.3.2.1. Methods—data collection

Housing and healthcare providers were recruited using e-mail and personal invitations. Enrolled participants met the following inclusion criteria: (1) current employment at the local Health Care for the Homeless or another clinic serving the majority of low-income patients, shelter, or social service agency serving people experiencing homelessness or unstable housing; (2) at least 12 months of experience; and (3) speak English. There were no listed exclusion criteria, and we took care to avoid coercion in recruitment methods. A trained research staff person (LJ) who had no role in the clinic or housing systems completed the interviews between June 2019 and March 2020, following a structured interview guide in-person and in private offices or conference rooms in or near their workspaces. Providers were offered a gift card of $5 from a local coffee shop as a modest compensation.

Similar to patient participants, we asked providers to comment on the following aspects of the planned treatment: use of BA, number, duration, and format (in-person, phone, or virtual) of intervention sessions, what to call the interventionist, and potential health behavior change goals. In addition, we specifically asked providers about the alignment of the BA approach to person-centered and harm reduction philosophies of care, how to best coordinate the intervention within existing systems, and currently available tools and resources to support health behavior change and suggested commercial tools to support health behavior change.

##### 2.3.2.2. Methods—data analysis

We followed identical procedures to patient transcripts as summarized above.

##### 2.3.2.3. Results

Individual interviews with providers (*N* = 21) were conducted with 14 clinic-based providers (7 nurses/nurse practitioners, 3 doctors, 3 social workers, and 1 pharmacist) and 7 housing or outreach providers (1 street outreach, 5 housing advocates) from July to December 2019. Providers identified as white (65%) and Black/African American (29%) and most identified as female (86%). Providers had a mean of 10 years experience (range 3–30 years) and estimated that they worked with a mean of 14 people with type 2 diabetes each week (range: 2–87.5) (see Supplementary Table 2).

Providers reinforced many of the findings from the patient participants including:

1. The need to take an integrated behavioral approach to mood/wellness and diabetes self-care and medication adherence. One provider noted, “*most of these people that have diabetes are also experiencing at least eight other co-occurring health and mental health conditions if not substance use disorder as well, so it's very complex*.”
2. They also reinforced the complexity and stigma surrounding homelessness, diabetes, and substance use faced by patients with some providers reinforcing patient fears about presuming intravenous drug use by those possessing needles. Another provider explained, “*And whether it's substance use getting in the way of eating properly, taking medicines, remembering to take medicines and come to appointments. Or patients are sometimes very afraid to take their medicines if they're using, like 'What if I mix this or that? I don't want to mess something up.' So, it's very common that [taking medications] goes to the wayside when people are actively using.”*
3. Providers suggested the interventionists *not* be called “case managers” to avoid overlap with other existing roles on the team or misperception that they would provide housing. Furthermore, they detailed the characteristics needed in a good interventionist who could work well with this population. These included being “*accommodating, open-door*,” “*blameless, blame free* [of participants],” and should have “*a tenacity to sort of stick with people even if they have a bad day or a bad interaction*.” One American Indian provider emphasized the need to “*find someone who's been there, find a Native person. Or find someone who has worked in that community*.” A physician described “*You need an incredibly talented person who knows how…to not just connect people with agencies, but help walk them through it*,” the “*kind of person…who can [garner] trust and who can recognize some of the barriers that the rest of society doesn't even see*.”
4. Providers also emphasized the importance of housing on intervention timing and participant readiness. As one told us, “*There's just so many things that are missing if you don't have housing.”* One physician described a specific patient who became angry and asked, “*Why can't you doctors control my diabetes?*” They told the patient, “*Once you get housing, we'll get it…controlled*.” This motivated the patient to talk to their housing support worker who we also interviewed.
5. Providers also had divided opinions about whether the intervention should focus on medication adherence alone vs. all diabetes self-care. They noted similar concerns to those of the patient participants, “*Diet and exercise is challenging when you're homeless. Because a lot of times…you can only do what is within your limits.”* Another provider noted that when you're homeless you're often walking around a lot and do not need extra exercise. Others noted the importance of addressing lifestyle “*because no matter how much medications we throw at people, it's not going to help unless we're able to address some of those things [diet and exercise].” One* provider connected mental healthcare to diabetes care telling us, “*Well, they have to be incorporated, all of them, medication, exercise, and diet, in order for their diabetes to get better. It's a combination of things and self-care, mental care should be addressed too.”* One provider emphasized the value of sequenced goals starting with medication adherence and then moving on to diet and exercise. “*Don't throw everything at once”*, if you do, she warned “*that's overwhelming…people can't handle all that.”*

Patient and provider data aligned on many other decisions about how to adapt the D-HOMES intervention to participants' realities (Supplementary Table 3). They reinforced our planned behavioral approach, refined our approach to diabetes education, and emphasized how to integrate D-HOMES into existing housing and healthcare services. Specific feedback supported the use of BA in its focus on personalized goal setting and focus on additional support from someone they could trust about diabetes. Specific elements of planned treatment decisions were reinforced (offering 10 one-on-one sessions over 12 weeks and delivering the intervention in convenient locations for participants). While other decisions were newly considered, e.g., naming the interventionist, input from participants and providers along with the evolving COVID-19 pandemic reinforced our decision to offer in-person, phone, and virtual participation options after an initial in-person assessment visit and the first coaching session.

#### 2.3.3. Community-engaged research team impact

The Quorum team had a substantial impact on Step 2 activities. This began with adapting the methods for the qualitative interviews. The team offered feedback to improve the clarity of the informed consent document and the patient question guide. They also completed the eco-map activity and advised the adaptation to use it to guide the entire interview (rather than as an individual warm-up activity). The team supported participant recruitment efforts directly and indirectly: This included directly hosting and assisting with participant identification for two focus groups at the downtown library and a third at a local American Indian community center, thus improving the diversity of patient participants. They also indirectly encouraged researchers to distribute flyers for focus groups via relationships with trusted healthcare and housing partners. Finally, the team brainstormed names for the interventionist after the interview data discouraged the use of “case manager.” The team voted to approve “Diabetes Wellness Coach” as the formal name used for the D-Homes interventionist.

Furthermore, we removed the Brief Trauma Questionnaire from assessments as recommended by a research staff person with a personal history of homelessness and work in homeless services who observed participant distress multiple times during Step 2 survey completion. This decision was made specifically to not alienate potential participants with a history of trauma at baseline in Step 3. Our team was clear that it was important to be sensitive to trauma history and trauma responses in the D-HOMES manual, and we integrated trauma into staff and coach training.

## 3. Step 3: open trial

### 3.1. Final D-HOMES treatment manual

**Figure 2:**
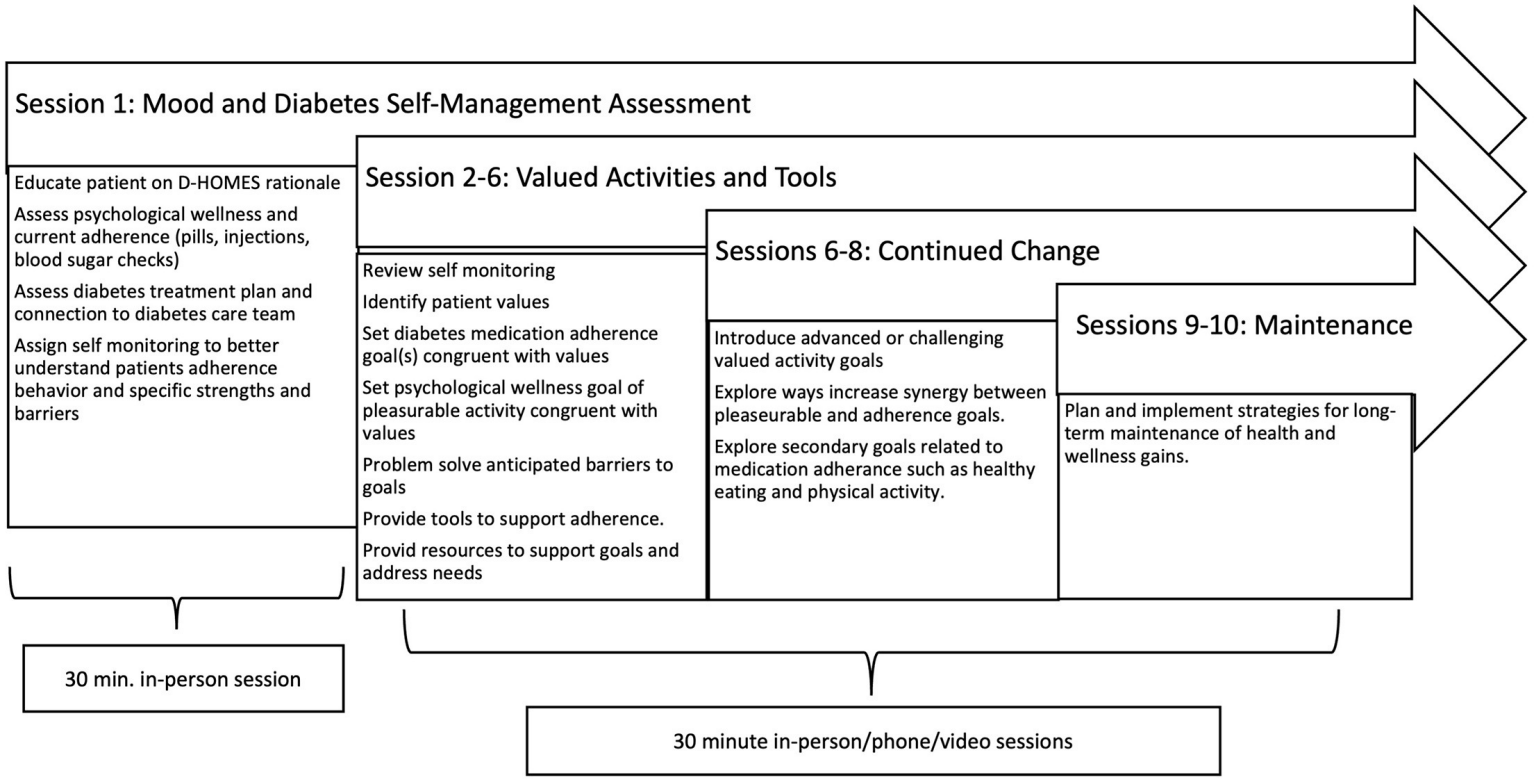
Diabetes Homeless Medication Support (D-HOMES) treatment session content.

Synthesizing findings from Steps 1 and 2 and ongoing input from the participatory research team, we finalized treatment session content to integrate evidence-based diabetes and BA interventions (Busch et al., 2015; Gathright et al., 2022; Adkins-Hempel et al., 2023). Session content is outlined in Figure 2 and followed a standard BA sequence. Treatment began with an introductory in-person session (1) where coaches outlined the treatment rationale, built trust, and assessed participants' current diabetes treatment plan and care team. Daily self-monitoring deepened the coach and participant's understanding of current strengths and barriers to medication adherence in session 2 and reinforced the idiographic interaction of psychological wellness and medication adherence. Coaches used patient-centered motivational interviewing techniques to support participants who expressed ambivalence regarding medication adherence. We offered sessions 2–10 in-person, or by phone or video per participant preference. Session 2 also involved a structured form to identify patients' life values. Coaches then introduced values-congruent goals with at least 1 goal aligned with diabetes medication adherence and 1 goal to enhance psychological wellness focused on pleasurable activities. Aligned with BA principles, coaches supported patients to define goals that were specific, measurable, appropriate, relevant, and time-stamped (SMART). Coaches guided participants to anticipate and problem-solve any barriers to planned goals (e.g., difficulty remembering medications might prompt setting an alarm on a patient's cell phone). Sessions 3–6 assessed progress toward diabetes medication adherence and wellness goals and used BA techniques to explore any internal or external barriers that impeded goal achievement since the previous session. Coaches supported participants to revise, sustain, or advance goals as appropriate. Participants received written copies of goals after each session and maintained contact with their coach by their preferred form of communication (email, text, calls, etc.) between sessions. Coaches guided participants to choose one commercially available tool to support their goals (e.g., notebook to record glucose levels or 3-time/day pillbox) and any needed resources to meet material needs (e.g., drop-in centers with hot meals and/or discount gym memberships). Sessions 6–8 introduced advanced goals for appropriate participants who demonstrated mastery of initial goals. If desired by the participant and deemed appropriate by the coach, this included moving from medication adherence to healthy eating or physical activity. Sessions 9–10 also included reflection on the program and engaged participants in planning for the maintenance of healthy behaviors developed during coaching. As physical or behavioral health needs emerged during sessions, coaches explored current or historically used services (providers and clinics) and provided resources tailored to participants' preferences (e.g., available primary care clinics or mental health professionals available for walk-in vs. scheduled visits per participants' preferences). Frequently coaches included exploring and initiating the use of such resources as SMART goals to encourage and support resource use.

### 3.2. Interventionist description and training

The PI (KDV, a licensed primary care physician with advanced training in behavioral medicine) provided coaching for the first 2 participants with consultation from a licensed clinical psychologist (AB) to serve as case examples for subsequent coaches. Non-expert providers (ES and JC) then completed the remaining coaching sessions. They included one bachelor's level social worker and one bachelor's level exercise physiologist. They underwent 22 h of training in total with 12 h devoted to self-study, didactics, discussion, and role-play in BA and basic motivational interviewing (overseen by AB). We included an emphasis on trauma-informed care and how to approach comorbid psychological distress, psychiatric diagnoses, and substance use. Diabetes training consisted of 10 additional hours of training in the Association of Diabetes Care and Education Specialists' 7 self-care behaviors of healthy coping, healthy eating, being active, taking medication, monitoring, problem-solving, and reducing risks (Tools and Resources for Living With Diabetes, n.d.). Furthermore, we reviewed guideline-based diabetes care conducted using the *Standards of Diabetes Care* (American Diabetes Association Professional Practice Committee, 2021), and educational handouts (Learning About Diabetes, n.d.) recommended by staff from a diabetes trial involving community health workers (Two Feathers et al., 2005). We took care to ensure that coaches knew how to support participants to recognize the symptoms of hypoglycemia, trauma, and/or suicidality and created safety protocols to guide coaches to connect participants to existing care teams and resources and/or receive support from the PI as needed. Resource and care coordination training consisted of didactics and familiarization with local referral sites including the local safety net hospital, Health Care for the Homeless Program, and area mental health and substance use resources especially those with walk-in or other easily accessible services. We conducted further training on medical racism and stigma influenced by the content of the qualitative interview data and the Quorum team. Interventionists attended numerous community-engaged research team meetings and completed mock treatment sessions with interested team members in addition to mock sessions with other lab staff before working with research participants.

### 3.3. Trial design

We designed a 12-week open (single-arm) trial focused on the acceptability and feasibility of D-HOMES. Recruitment methods included referral from housing and healthcare providers; invitations to interested patient participants in Step 2 qualitative interviews; letters and follow-up phone calls to safety net health system patients meeting inclusion criteria from data in their electronic medical records; flyers at shelters, libraries, and homeless drop-in centers; and snowball recruitment from enrolled participants and Quorum team members. Inclusion criteria were: (1) age 18 years or older, (2) English-speaking, (3) homelessness by federal definition in the last 12 months, (4) self-reported diagnosis of type 2 diabetes, later verified in the medical record, (5) hemoglobin A1c >7.5%, (6) plan to stay in the local area or be reachable by phone for the next 16 weeks, and (7) willingness to work on medication adherence and diabetes self-care. Exclusion criteria were (1) inability to provide informed consent (e.g., presence of a legal guardian and prisoners), (2) active psychosis or intoxication limiting the ability to give informed consent, and (3) pregnant or lactating females. Participants were offered incentives at assessment visits ($10 and $20 at 2 baseline visits and $45 at post-treatment visit), weekly support for travel costs, payment for phone minutes ($10/week for up to 12 weeks), and a $20 final bonus payment for a maximum total of $215.

Before recruitment began, we registered the pilot trial with ClinicalTrials.gov (NCT04678284; Vickery, 2023).

Recruitment began with an initial phone screen to assess some inclusion/exclusion criteria. Eligible participants were then invited for baseline visits to complete consent and eligibility lab testing over two visits. Participants then received up to 10 coaching sessions over 12 weeks as detailed above (See above, 3.1 Final D-HOMES treatment manual).

We monitored coaching content via a structured checklist (fidelity form) used by all coaches to ensure coverage of similar intervention content. All coaching sessions were audio recorded. Coaches connected with the PI and/or mentor (AB) during weekly supervision. The PI (KDV) and mentor (AB) reviewed all audio recordings and provided feedback during these meetings. When components of the structured checklists were not met, we gave in-depth feedback including suggested phrasing and role play to enhance fidelity to the treatment manual.

We completed a final assessment visit 12 weeks after coaching began. The primary outcome of intervention acceptability was the Client Satisfaction Questionnaire (CSQ, 8-item version). Furthermore, non-coaching staff conducted post-treatment interviews at the final assessment to ask for input about coaching content, delivery format, and trial procedures. We also collected secondary outcome measures to demonstrate measurement feasibility for a future randomized trial: Hemoglobin A1c (A1c, using fingerstick point-of-care machines), medication adherence [diabetes-specific (ARMS-D; Mayberry et al., 2013)], overall (ASK-12; Matza et al., 2009), diabetes self-care (DSMQ; Schmitt et al., 2013), diabetes distress (PAID-5; Polonsky et al., 2005), substance use [adapted WHO ASSIST questions (Smith et al., 2010; NIDA Quick Screen V1.0., n.d.)], and psychological wellness (SF-12; Johnson and Coons, 1998 and MHI-5; Rumpf et al., 2001), housing stability, competing demands (Basic Needs Survey; Gelberg et al., 1997), and care coordination (questions about diabetes and overall healthcare use).

We used appropriate univariate statistical methods to summarize survey responses (e.g., CSQ-8) and paired *t*-tests to estimate the change between baseline and post-treatment assessments for A1c results and survey items asked at both time points. We audio-recorded all post-treatment interviews. We used framework matrix analysis to summarize content from the audio recordings (Gale et al., 2013) (similar procedure to above, see 2.2.1.2). The analysis focused on participants' satisfaction and critiques, and suggested changes to the planned intervention.

### 3.4. Pilot trial results

#### 3.4.1. Recruitment and enrollment

**Figure 3:**
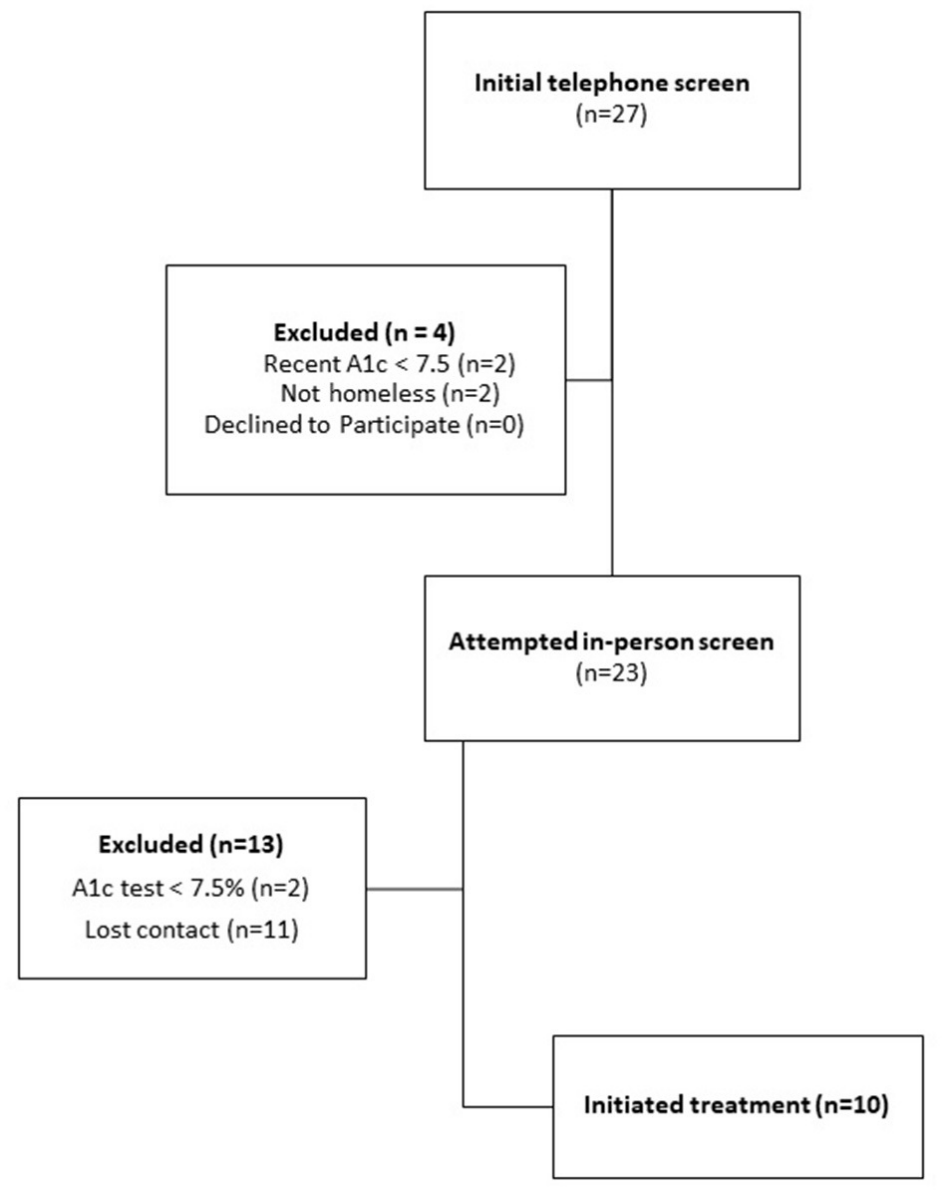
Diagram of open trial participant screening, enrollment, and treatment completion.

We reached 27 potential participants for the initial phone screening. This yielded 23 potentially qualified participants, and 10 final participants who initiated treatment (43% uptake) (Figure 3). Among screened participants who did not enroll, the majority lost contact with study staff (*n* = 11).

Despite our emphasis on hiring staff of color and involving diverse members of the Quorum team in the study design, we noted a racial pattern in screened participants and their likelihood to enroll (data not shown): Four of 5 screened white participants enrolled while only 5 of 11 screened Black participants enrolled (1 had a low A1c and 6 lost contact); and 1 of 5 screened American Indian participants enrolled (2 had low A1c and 2 lost contact).

Table 3 summarizes the characteristics of trial participants (*N* = 10). A majority identified as male gender and half as Black race. While participants took an average of 7.3 (SD 3.8) total medications, 3 participants (30%) took only oral diabetes medications, 1 participant (10%) took only insulin, and 6 (60%) took both for diabetes. Housing at enrollment varied among participants with 4 living in their own apartment or house. However, based on other enrollment questions, these locations often involved subsidized housing, and a majority of participants expressed worry about not having housing in the next 2 months. Participants had high rates of physical and behavioral health comorbidities and tobacco use but few reported use of other substances. Two participants (20%) reported being diagnosed by a provider with PTSD.

**Table 3:** Demographic and related baseline characteristics of open trial participants.

**Demographic characteristics, *n* (%) or otherwise specified**	***N =* 10**
Age (mean years, SD)	57.40 (4.12)
Gender, men	6 (60.0)
Race
Black	5 (50.0)
American Indian	1 (10.0)
White	4 (40.0)
Hispanic Ethnicity	1 (10.0)
Education	
Less than high school	1 (10.0)
High school graduate/GED	3 (30.0)
Some college/technical degree/Associate degree	4 (40.0)
College graduate (BA or BS)	2 (20.0)
Number of prescribed medications (mean, SD)	7.30 (3.80)
Health insurance	
Medicaid or MinnesotaCare	3 (30.0)
Medicare	4 (40.0)
Other	3 (30.0)
Housing at enrollment	
Homeless shelter	2 (20.0)
House/apartment of relatives or friends	1 (10.0)
In own house/apartment	4 (40.0)
Outdoors (street, park, beach, tent)	1 (10.0)
Residential treatment for drug or alcohol use	1 (10.0)
Transitional housing	1 (10.0)
Worried about not having stable housing in the next 2 mo. (yes)	6 (60.0)
Co-morbidities (*n*, %)	
High blood pressure	7 (70.0)
High cholesterol	6 (60.0)
Depression	6 (60.0)
Anxiety/panic disorder	5 (50.0)
Arthritis	4 (40.0)
Heart disease	5 (50.0)
Asthma	2 (20.0)
Emphysema or COPD or chronic bronchitis	1 (10.0)
Liver problems	1 (10.0)
Post-traumatic stress disorder (PTSD)	2 (20.0)
Traumatic brain injury (TBI)	1 (10.0)
Bipolar disorder	1 (10.0)
Schizophrenia/schizoaffective	0 (0)
Body mass index (mean, SD)	33.7 (7.3)
Reported substance use, any (*n*, %)	
Tobacco	6 (60.0)
Alcohol	1 (10.0)
Cannabis	2 (20.0)
Amphetamine	1 (10.0)
History of overdose (lifetime) (*n*, %)	1 (10.0)

#### 3.4.2. Feasibility data: engagement and retention

The 10 participants who initiated treatment demonstrated moderate engagement with an average of 6.4 coaching sessions completed over 12 weeks (median = 7, mode=10, range 1 to 10, SD=3.7, Table 4). During weekly coaching sessions, participants set an average of 3.1 BA goals to work on between sessions (SD 1.3, range 0 to 6) and self-reported achieving 76% of these goals. Nine of 10 participants completed some component of the final assessment. Eight participants completed the CSQ (primary outcome) and 7 completed a valid post-treatment A1c (planned primary outcome for follow-up trials).

**Table 4:** Feasibility and acceptability data and A1c change among open trial participants.

**Pt.N**.	**Housing at enrollment**		**Number of coaching sessions**	**Acceptability (CSQ-8)**	**Post-tx interview done**	**Baseline A1c**	**Post-tx A1c**	**Change in A1c**
1	More stable	House/apartment	10	31	Yes	10.4%	8.5%	−1.9%
2		Transitional housing	10	32	Yes	9.6%	9.4%	−0.2%
3		House/apartment	10	32	Yes	8.5%	6.1%	−2.4%
4		House/apartment	10	32	Yes	12.1%	11.4%	−0.7%
5		House/apartment	7	32	Yes	8.1%	9.8%	+1.7%
6	Less stable	Shelter	7	16	Yes	7.7%	7.3%	−0.4%
7		Friend's house/apt.	5	8	Yes	11.4%	12.8%	+1.4%
8		Outdoors	2	N/A	Yes	10.7%	N/A	-
9		Shelter	1	20	No	12.1%	N/A	-
10		Substance use tx.	2	N/A	No	10.1%	N/A	-
*N =* 10			Mean = 6.4	Mean = 23.4	*n =* 8			Mean^*^= −0.4%

N/A, Not assessed. Study staff worked with participants to encourage as many post-treatment assessments as possible while respecting autonomy and participants' preferences.

* Mean change includes only 7 participants who completed baseline and post-treatment HbA1c measurements.

Engagement in coaching sessions and assessments and retention varied with housing status (Table 4). Five participants with the most stable housing completed more coaching sessions (with most completing all 10 and 1 participant completing 7 sessions). These participants also completed most items in the final assessment Five participants with less stable housing had fewer coaching sessions (1, 2, 2, 5, and 7 sessions). They also completed fewer post-treatment assessments with 1 providing no post-treatment assessment data (participant #10) and overall 2 missing CSQ scores, 2 missing post-treatment interviews, and 3 missing post-treatment A1c measures.

#### 3.4.3. Acceptability data

Overall, 8 participants completed the CSQ. Participants rated D-HOMES with a mean score of 23.4 out of 32 possible points (range 8 to 32 with high points indicating more satisfaction). Acceptability also varied with housing status (Table 4). More stably housed participants had consistently higher scores (*n* = 5, mean = 31.8, range 31–32, SD = 0.4; indicating high satisfaction with the intervention). Less stably housed participants who responded to the CSQ (*n* = 3) had lower scores (mean 14.7, range 8–20, SD = 6.1; indicating low satisfaction with the intervention).

We further assessed acceptability with post-treatment qualitative interviews with 8 participants. Again, responses confirmed our pattern of variation by housing status with more stably housed people reporting more favorable feedback and less stably housed people reporting more critiques. The 3 less stably housed participants who participated in interviews had strong critiques of the program that shaped treatment adaptation: One participant felt we had poorly explained the study and did not understand the timeline for longitudinal coaching and assessment. The second participant moved as she enrolled in the study and completed one coaching session. She appreciated the incentives but explained, “*There's no way I can stick to any of the plans I've been given by a doctor…because my life is chaotic. Too many people calling me too many people texting me; too much to do. I've had to cut back and say forget it. Some of this has got to go away*.” A third participant who completed 7 sessions stayed in the shelter and had numerous chronic physical and mental health conditions. She described numerous interpersonal conflicts with other professionals and reported she “*didn't find the coach to be helpful*” and was upset about a rescheduled session and the time it took for her to receive research incentives. However, despite her overall negative impression, she noted positives that the coach supported her to accomplish goals, taught her to use her glucometer using videos (her preferred learning modality), and provided her with a tailored tool that she liked (a glucose logbook with a floral pattern). She also noted her appreciation for the mix of in-person and phone-based sessions, coach stability, and the 12-week time frame.

The 5 more stably housed participants expressed strong support for the D-HOMES program. They cited the coach's accountability, honesty, enthusiasm, resource sharing, and support to overcome barriers as contributing to this conclusion. One noted he “*got a lot accomplished here in a short time*” and especially valued the emotional support that he was not getting from other people in his life. Participants liked the process of personalized goal setting and learning how to set appropriately scaled goals, and at least one described plans to apply this skill to other areas of life after treatment. Participants appreciated the tools to support behavior change and the resources recommended by the coach. All these participants liked the integration of psychological wellness with behavior change goals. As one participant shared, “*It's hard to feel good physically when you don't feel good mentally*.” Three participants preferred in-person meetings, and two appreciated a mix of phone and in-person. Most participants described the length and number of sessions as “*just right*,” and several noted that time passed quickly. One participant noted that he'd like to “*double the length*” of the support for his behavior change. Participants described the compensation as appropriate and important.

#### 3.4.4. Clinical outcome data

**Figure 4:**
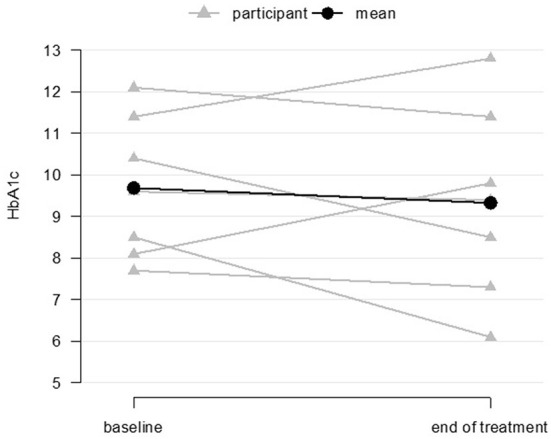
Glycemic control at baseline and end-of-treatment for coaching participants (*n* = 7 baseline and end-of-treatment).

The planned, fully powered D-HOMES RCT will be powered on the primary outcome of glycemic control as measured by hemoglobin A1c (A1c). In this pilot, 7 of 10 participants (70%) had both a baseline and 12-week A1c value measured. Among that group, we found an overall change in the hypothesized direction toward improved glycemic control from baseline to post-intervention assessment (12 weeks) (Figure 4) [baseline mean = 9.7 (SD 1.7), post-treatment mean = 9.3 (SD 2.3), mean change= −0.4 (SD 1.5), *p* = 0.56].

Table 5 summarizes secondary outcomes between baseline and 12 weeks. We found inconsistent, non-significant changes in psychological wellness with worsening scores in physical and mental health on SF-12 measures but improved scores on the MHI-5 measure. We found a non-significant change in the hypothesized direction toward less diabetes distress (score changed from baseline to post-treatment −1.5 points, *p* = 0.66). Participants demonstrated minimal changes in self-reported medication adherence or diabetes self-management. BMI and diastolic blood pressure changed minimally, while systolic blood pressure increased by 14 mmHg (*p* = 0.04) from baseline to post-treatment.

**Table 5:** Psychological wellness, diabetes distress, medication adherence and self-management, and biometrics at baseline and end-of-treatment (*n* = 6–10).

	**Baseline (mean, SD)**	**Post-treatment (mean, SD)**	Change (SD)^*^	*p*-value^*^
	***N =* 10**	***n =* 6–7**	*n =* **6–7**	
**Psychological wellness (SF-12) (** ***higher scores mean better health/functioning)***				
Physical health (PCS-12)	36.6 (12.4)	31.6 (14.3)	−4.9 (8.0)	0.19
Mental health (MCS-12)	42.6 (14.1)	40.0 (13.3)	−4.2 (11.3)	0.41
Psychological wellness (MHI-5) (*higher scores mean more wellness)*	36 (26)	43 (28)	11 (26)	0.36
Diabetes distress (PAID-5) (*higher scores indicate more diabetes distress)*	9.9 (7.0)	7.5 (6.7)	−1.5 (7.8)	0.66
Diabetes medication adherence (ARMS-D) (*higher scores mean worse adherence)*	16.2 (3.0)	16.8 (3.8)	1.2 (3.5)	0.46
Medication taking subscale	9.4 (2.3)	10.0 (2.2)	0.3 (2.0)	0.70
Medication refill subscale	6.8 (2.4)	7.0 (1.7)	0.8 (1.8)	0.32
All medication adherence (ASK-12) (*higher scores mean more barriers to adherence)*	33.8 (6.3)	35.0 (5.1)	1.3 (4.2)	0.43
Health beliefs subscale	11.5 (2.6)	12.7 (3.5)	1.0 (3.0)	0.39
Inconvenience or forgetfulness subscale	7.6 (3.2)	6.8 (2.4)	−0.3 (3.1)	0.82
Behavior subscale	14.7 (2.2)	15.3 (3.9)	0.6 (3.8)	0.70
Diabetes self-management (DSMQ) (*higher scores mean better self-management)*	21.5 (6.9)	22.9 (6.1)	1.2 (3.4)	0.44
Glucose subscale	5.7 (2.5)	5.5 (2.8)	−0.2 (1.8)	0.78
Diet subscale	5.2 (2.5)	4.9 (2.2)	−0.8 (2.0)	0.35
Physical activity subscale	3.0 (3.2)	5.2 (2.0)	0.9 (3.7)	0.56
Healthcare use subscale	2.4 (2.4)	3.3 (1.9)	1.3 (1.9)	0.16
**Biometric measures**				
BMI (kg/m^2^)	33.7 (6.9)	31.9 (4.8)	−2.1 (2.7)	0.12
SBP (mmHg)	131 (16)	147 (15)	14 (13)	0.04
DBP (mmHg)	81 (11)	89 (14)	6 (12)	0.28

* Change and p-value from paired t-test comparisons included only participants who completed baseline and post-treatment measurements.

### 3.5. Community-engaged research team impact

Throughout Step 3, the Quorum team helped to manage the open trial. Before recruitment began, the team revised participant-facing treatment materials such as a values assessment worksheet used in D-HOMES. They changed the original language defining values to better reflect these sentiments in casual and approachable language: “*Values are the stuff that really matters to you, the things that you need to live a content life. Our values lead us to valued activities that help us lead our best life.”* They also adopted a handout outlining example values to better reflect their experiences, e.g., changing “family” to “family/friends,” and “work” to “work/volunteering,” and adding “financial stability” as a value that was missing from the original worksheet. The team also led revisions of the study logo (de-emphasizing housing to avoid the misperception that we provide housing) and adapted study recruitment materials (letters and flyers). During the trial, the Quorum team also helped adjust recruitment materials, with IRB approval, including adding efforts to better reach and retain people of color. The research staff and PI reviewed cases of lower engagement with the team, and the team brainstormed different responses to participants. Needed changes sometimes happened during the open trial and sometimes in the planned next step, a randomized pilot trial (NCT052586303), see the Discussion section below.

## 4. Discussion

We conducted a three-step process guided by a community-engaged research team to develop a behavioral lifestyle intervention for PEH with diabetes: the Diabetes Homeless Medication Support (D-HOMES) program. Our pilot test, a single-arm open trial enrolled 10 people, and we concluded that the D-HOMES intervention was feasible and acceptable to the target population. We found a similar degree of change in glycemic control (HbA1c) as other behavioral interventions and health coaching programs for diabetes (Medical Advisory Secretariat, 2009; Shetty et al., 2022). Other measures demonstrated inconsistent changes in baseline to post-treatment comparisons. With only 6–7 participants providing the baseline to post-treatment data, no conclusions regarding efficacy should be drawn. Lack of change in our target behavior of diabetes-specific medication adherence may relate to a ceiling effect. That is, despite all participants having poor glycemic control at baseline, *self-reported* adherence at baseline was quite high with little room for improvement (ARMS-D mean at baseline was 16.2; perfect adherence on the ARMS-D would score an 11, and worst adherence would score 44). Future work should explore the validity of the ARMS-D in this population.

One limitation of our study includes the lower enrollment rate among people of color. We plan several modifications to address this as we launch the next step, a randomized pilot trial comparing the D-HOMES intervention to a brief educational session: (1) We worked with our community-engaged research team and research staff with lived experience to make a recruitment video highlighting the diverse team who supported the work (The Diabetes Medication Support (D-HOMES) Program, 2022). (2) We added staff training about stigma and structural racism in housing using a local documentary highlighting the history of redlining in our region (Jim Crow of the North, 2021) and added a measure of racial discrimination in healthcare to our randomized pilot trial to better understand this impact on our target population (Peek et al., 2011). (3) We also adapted our enrollment procedures to more clearly describe participation expectations for this longitudinal trial. This included a revised coversheet for our informed consent document. The Quorum team provided input and approval of the document which uses an infographic-based visual summary of study activities and timeline (Supplementary Figure 1). (4) We also revised the consent quiz, which we administer before randomization, to more clearly emphasize study procedures. We also note that this trial took place in Minneapolis, MN, concurrent with the murder of George Floyd and subsequent protests and the ongoing COVID-19 pandemic both of which disproportionately impacted communities of color and may have led to higher-than-average reluctance to engage with healthcare research (Barbot, 2020).

Another limitation we observed was the consistent pattern that the D-HOMES participants who were more stably housed had an easier time enrolling, engaging in treatment, and retaining contact with our study team. We considered limiting participation in the randomized pilot trial to only people with more stable housing. However, given strong data documenting ongoing structural racism leaving people of color vulnerable to longer waits to receive transitional or supportive housing (Olivet et al., 2021), we elected to continue enrolling people with all forms of unstable housing. With guidance from the Quorum team, we made several key adaptations in addition to those described above to improve the design of our randomized pilot trial in response to this observation: (1) We developed relationships with housing providers who could help us target diverse but more stably housed people exiting shelters or other temporary housing facilities. We sought housing partners known to be respected for their engagement with communities of color. (2) We designed booster calls and a monthly incentive to encourage retained contact with participants in both the intervention and comparison arms. We also designed a visually appealing postcard to outline available incentives over time to emphasize clarity among research staff and participants about what compensation participants could expect at what time points (Supplementary Figure 2).

We plan to continue working with the Quorum team to respond to the data from the ongoing randomized pilot trial. During the open trial, the team developed a preferred process we use monthly to review ongoing recruitment/retention and any adverse or unexpected events during the randomized pilot trial. The randomized pilot trial data will guide us to the decision of whether to pursue a fully powered randomized trial in the future. Recruitment data as well as feasibility and acceptability data will continue to shape our approaches to the larger trial.

The research and Quorum teams share the concern about the experiences of people of color and the least stably housed participants in our trial to date. In addition to proceeding with the development of the D-HOMES intervention, we also hope to pursue alternative intervention designs to better understand and meet the needs of less stably housed participants who may be most likely to have experienced recent trauma. Our data emphasize that the success of this work will depend on building a well-resourced team that can spend a lot of time engaging and building trust with people with lived experiences to determine together how best to overcome the substantial challenges that exist to recruiting, retaining, and engaging with people experiencing the highest degrees of housing instability. Our experiences conceptualizing, refining, and pilot testing a behavioral program to support people with type 2 diabetes who have experienced homelessness illustrate the potential for community-engaged research methods to address health equity in populations with high rates of trauma using and following the ORBIT model of incremental behavioral treatment development.

## Data availability statement

The raw data supporting the conclusions of this article will be made available by the authors, without undue reservation.

## Ethics statement

The studies involving humans were approved by Hennepin Healthcare Research Institute Institutional Review Board. The studies were conducted in accordance with the local legislation and institutional requirements. The participants provided their written informed consent to participate in this study.

## Author contributions

KV led the study design, data collection, analysis and interpretation, drafted the article, and integrated feedback from co-authors. BF supported the study design and data collection, assisted with analyses, and offered critical review and final approval of the manuscript. ZB substantially supported the study design, data collection and analysis, and offered critical review and final approval of the manuscript. ES substantially supported data collection and offered a critical review and final approval of the manuscript. LG, SG, EA, VM, ML, JC, MH, and PO'C substantially supported the study design and data interpretation and offered critical review and final approval of the manuscript. ME substantially supported data analysis and interpretation and offered critical review and final approval of the manuscript. AB substantially supported the study design, data collection, analysis, and interpretation, and offered critical review and final approval of the manuscript. All authors contributed to the article and approved the submitted version.
