# Supplementary material for: The development and initial feasibility testing of D-HOMES: a behavioral activation-based intervention for diabetes medication adherence and psychological wellness among people experiencing homelessness
Source: Front Psychol. 2023 Sep 19;14:1225777. doi: 10.3389/fpsyg.2023.1225777 (PMC10546874; doi:10.3389/fpsyg.2023.1225777)
Supplement: Supplementary file 1 [file Table_1.docx]

Supplementary Material

Supplemental Table 1. Demographic characteristics and comorbidities of focus group and interview participants living with type 2 diabetes who had experienced homelessness

| **Demographics** | N=26 |
| --- | --- |
| Age, years | Mean 55.3  Range 39-82 |
| Female Gender, n (%) | 11 (42%) |
| **Race, n (%)**  Black/African American  American Indian  White  Asian  Native Hawaiian or Pacific Islander  Other | 16 (62%)  4 (15%)  4 (15%)  1 (4%)  1 (4%)  3 (12%) |
| **Hispanic Ethnicity, n (%)** | 4 (15%) |
| **Educational attainment, n (%)** Less than high school  High school graduate/G.E.D.  Some college/Technical or Associates Degree  College graduate  Advanced degree  Missing | 4 (15%)  9 (35%)  10 (38%)  0  1 (4%)  2 (8%) |
| **Number of prescribed medications (mean, SD)** | Mean 8  Range 2-20 |
| **Diabetes medications, n, (%)** Insulin and oral medications  Oral medications only  Neither  Missing | 13 (50%)  9 (35%)  3 (12%)  1 (4%) |
| **Has known diabetes doctor/provider, n (%)** | 23 (88%) |
| **Co-morbidities, diagnosed by provider**  High blood pressure  Depression  High cholesterol  Anxiety disorder/panic  Heart disease  Post-traumatic stress disorder (PTSD)  Arthritis  Emphysema/COPD  Bipolar disorder  Traumatic brain injury  Asthma  Schizophrenia/schizoaffective disorder  Liver problems | 18 (69%)  18 (69%)  14 (54%)  14 (54%)  8 (31%)  8 (31%)  7 (27%)  5 (19%)  5 (19%)  4 (15%)  2 (8%)  2 (8%)  0 |
| **Reporting PTSD symptoms (BTQ)** Yes  No  Missing | 18 (69%)  6 (23%)  2 (8%) |
| **Substance use, any in past 30 days** Tobacco  Alcohol  Marijuana  Cocaine  Methamphetamine  Heroin  Hallucinogens  Inhalants  Prescription medication misuse  Other  Missing | 20 (77%)  7 (27%)  6 (23%)  2 (8%)  1 (4%)  1 (4%)  0  0  0  0  4 (15%) |
| **Housing at time of interview** A shelter  An apartment  No steady place to sleep at night  Car  Living with friends/family  Room/rented room  A transitional living situation  Residential treatment/supervised housing  Missing | 9 (35%)  6 (23%)  3 (12%)  2 (8%)  2 (8%)  2 (8%)  1 (4%)  1 (4%)  1 (4%) |
