# Supplementary material for: The development and initial feasibility testing of D-HOMES: a behavioral activation-based intervention for diabetes medication adherence and psychological wellness among people experiencing homelessness
Source: Front Psychol. 2023 Sep 19;14:1225777. doi: 10.3389/fpsyg.2023.1225777 (PMC10546874; doi:10.3389/fpsyg.2023.1225777)
Supplement: Supplementary file 2 [file Table_2.DOCX]

**Supplemental Table 2. Demographic characteristics of housing and health care provider interview participants**

| **Demographics** | N=21 |
| --- | --- |
| Age, years | Mean 41  Range 25-58 |
| Female gender, n (%) | 18 (86%) |
| **Race** White  Black/African American  American Indian  Asian | 15 (71%)  6 (29%)  1 (5%)  1 (5%) |
| **Ethnicity** Hispanic or Latino  Missing | 1 (5%)  3 (14%) |
| **Job title at time of interview** Nurse  Physician  Social Worker  Pharmacist  Street Outreach worker  Housing Advocate  Unemployed | 7 (33%)  3 (14%)  3 (14%)  1 (5%)  1 (5%)  5 (24%)  1 (5%) |
| Years in role serving people who are homeless | Mean 10  Range 3-30  SD 8 |
| Average no. of people with type 2 diabetes and homelessness served per week | Mean 14  Range 2-87.5  SD 22 |
| Average hours/week working with people with type 2 diabetes and homelessness | Mean 11  Range 1-56  SD 14 |
