# Supplementary material for: The development and initial feasibility testing of D-HOMES: a behavioral activation-based intervention for diabetes medication adherence and psychological wellness among people experiencing homelessness
Source: Front Psychol. 2023 Sep 19;14:1225777. doi: 10.3389/fpsyg.2023.1225777 (PMC10546874; doi:10.3389/fpsyg.2023.1225777)
Supplement: Supplementary file 3 [file Table_3.docx]

**Supplemental Table 3.** **D-HOMES program decisions based on formative qualitative interview data**

| **Intervention decision** | **Theme** | **Exemplary quotes** |
| --- | --- | --- |
| Need for a behavioral approach | Desire for mood and behavior support | *“I know all the rights and wrongs. I just – just doing the rights and wrongs* [presents difficulties for me]*.”*  *“I think, in addition to everything that we discussed, we would need to help a person deal with their feelings a little bit. Because we hide a lot, ya know? Not many people don’t know whatever we’re going through. And me, personally, I think someone to help you in dealing with the feelings, it’s…the critical stuff.”*  Providers noted the common presence of mental illness and/or substance use which can complicate diabetes care. One said, “*most of these people that have diabetes are also experiencing at least eight other co-occurring health and mental health conditions if not substance use disorder as well, so it’s very complex.*” |
|  | Interaction of mood and medication adherence | *“Well, a lot of times too, when you get stressed out you also get depressed. And when I get depressed, I don’t take my medication. I don’t do anything”*  *“I have, like, unstable moods and, like, impulsive behavior. …[This impacts my diabetes] by, like, just not thinking clearly and just don’t take your medicine and eat right”*  Providers reinforced this observation. One noted *“You see a lot of hopelessness…Just a lot of ‘whatever, I don’t even care [my future or about taking my medications] no more.’”* |
|  | Interaction of mood with eating choices and diabetes | *“When you don’t have options and choices, of course you’re going to feel bad. I tend to overeat [in these situations].”*  Providers also noted the impact of stress on eating and, in turn, diabetes. |
| Diabetes education | Confusion areas about diabetes | *“I want to know more about my meds, all the meds I’m taking and I’m taking a lot of meds.”*  Several providers noted mis-information and poor understanding of diabetes especially about medications and the use of glucometers. |
| Offering materials in numerous learning formats | General intervention response | *[Preferred learning style] “For me it would be video” citing limited education and literacy*  *“But brochures I like.”* or printouts or paper with need for easy-to-read options *“I learn [by] verbal or written.”*  Other participants requested materials via email or text, website or video, and hands on learning. Providers emphasized their need to be creative and use pictures and hands on demonstration to achieve health education. |
| Coordination with existing services (mental health, visiting nurses, housing case managers) | Strategies for success in medication adherence  General intervention response | Providers noted that people were focused *“fundamentally [on] survival.”* And described *“on top of the hustle and bustle of trying to get out of homelessness, be it meeting with social workers, case managers, [Assertive Community Treatment] teams, or fending for oneself, applying for apartments, and just trying to leverage their way out of homelessness, diabetes, unfortunately, takes a deep backseat.”*  *“Because you can think you’re doing a lot by, kind of, having all of these supports in various ways. But if nobody is aware of what the other people are doing? I guess it seems like that coordination piece…would be really key.”* |
| Behavioral activation, use of personalized goals aligned with values and accountability | General intervention response | *“I don’t want nobody to just come in and listen to me talk…I need somebody that can help me keep on being independent.”*  Providers acknowledged celebrations of small successes are often crucial to building trust and supporting health behavior change.  Another provider emphasized *“You know, every client is different…You just have to approach them, differently”*  And one emphasized the need to focus on incremental, appropriate goals *“If you are asking someone to set a goal that they are incapable of, then we’re not helping them.*” |
| Avoidance of traditional mental health terminology | General intervention response | *“I don’t want no…psychologist where the only thing I’m doing is talking.”* |
| Connection to community resources | General intervention response | *“Somebody that can tell me about resources. ‘Hey [name], maybe this can help you?’”* |
| Relationship-centered | General intervention response | *“It does make a difference. It does make a helping hand to say, ‘Hey, you’re not alone. We’re thinking about you too.’”*  *Numerous housing and health care providers emphasized the need to start by building a trusted relationship.* |
| Emphasis on independence, accountability | General intervention response | *“Nobody doing it for me, but put me where that I know* [the next] *step and they making sure that it’s done and stuff like that.”*  *“To give that person the benefit of the doubt. You listen to that person, help them make choices and give them choices they can make on their own. Instead of just dictating.”*  *“So a case manager would be nice. Somebody to keep you on track, to kind of, remind you.”* |
| Suggested tools to support behavior change | Intervention tool suggestions | **Patients and** **Providers supported various tools**  Pillboxes  Notebooks/calendars  Bags  Diabetes medical alert bracelets  Hygiene kits with soap, hand sanitizer, lotion  Socks  Vouchers for diabetic shoes  Coolers  Glucose tablets  Snacks, food, food gift cards  Water/water bottles |
