# Supplementary figures and images for: The development and initial feasibility testing of D-HOMES: a behavioral activation-based intervention for diabetes medication adherence and psychological wellness among people experiencing homelessness
Source: Front Psychol. 2023 Sep 19;14:1225777. doi: 10.3389/fpsyg.2023.1225777 (PMC10546874; doi:10.3389/fpsyg.2023.1225777)

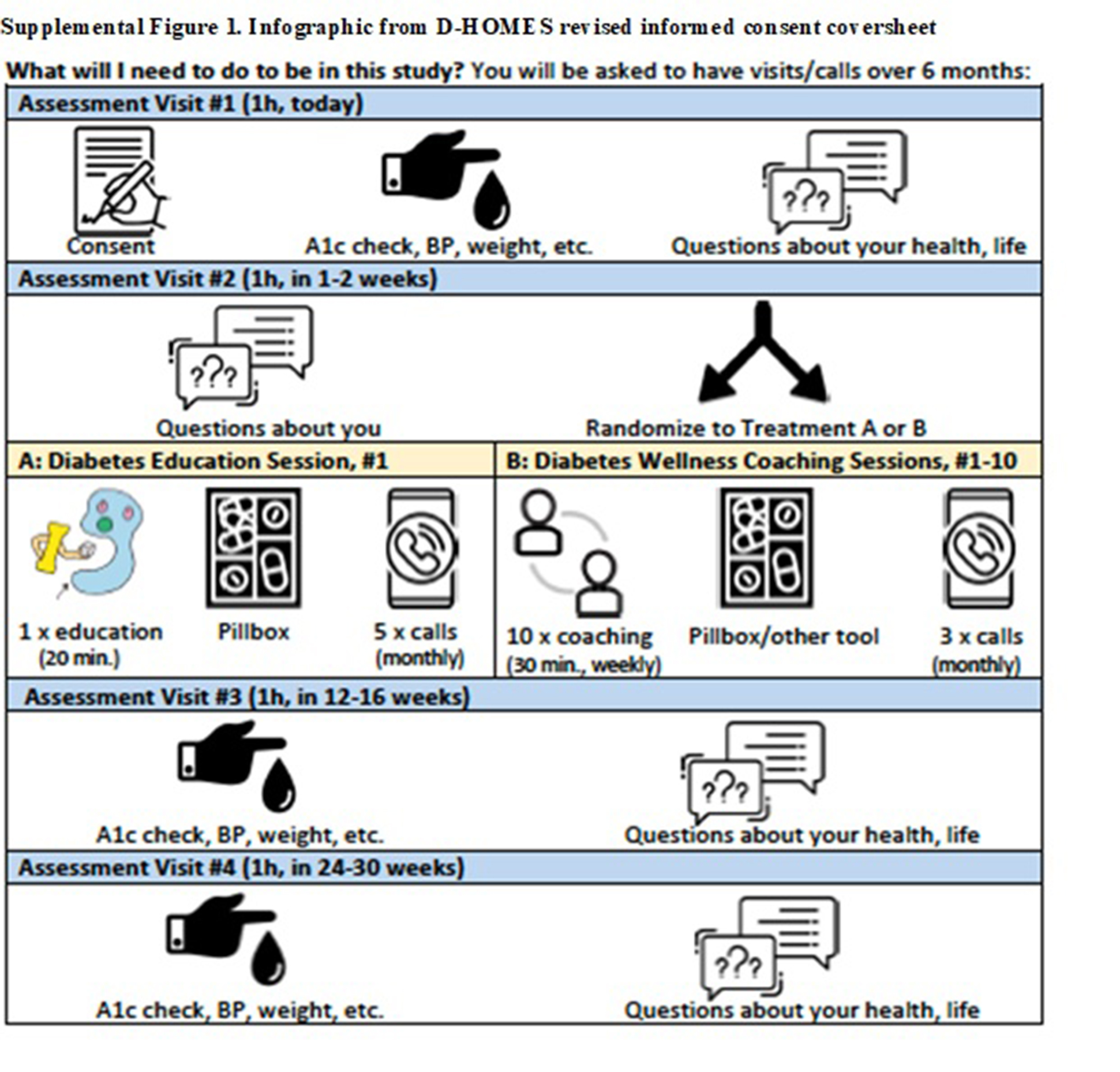

Supplement: Supplementary file 4 [file Image_1.JPEG]

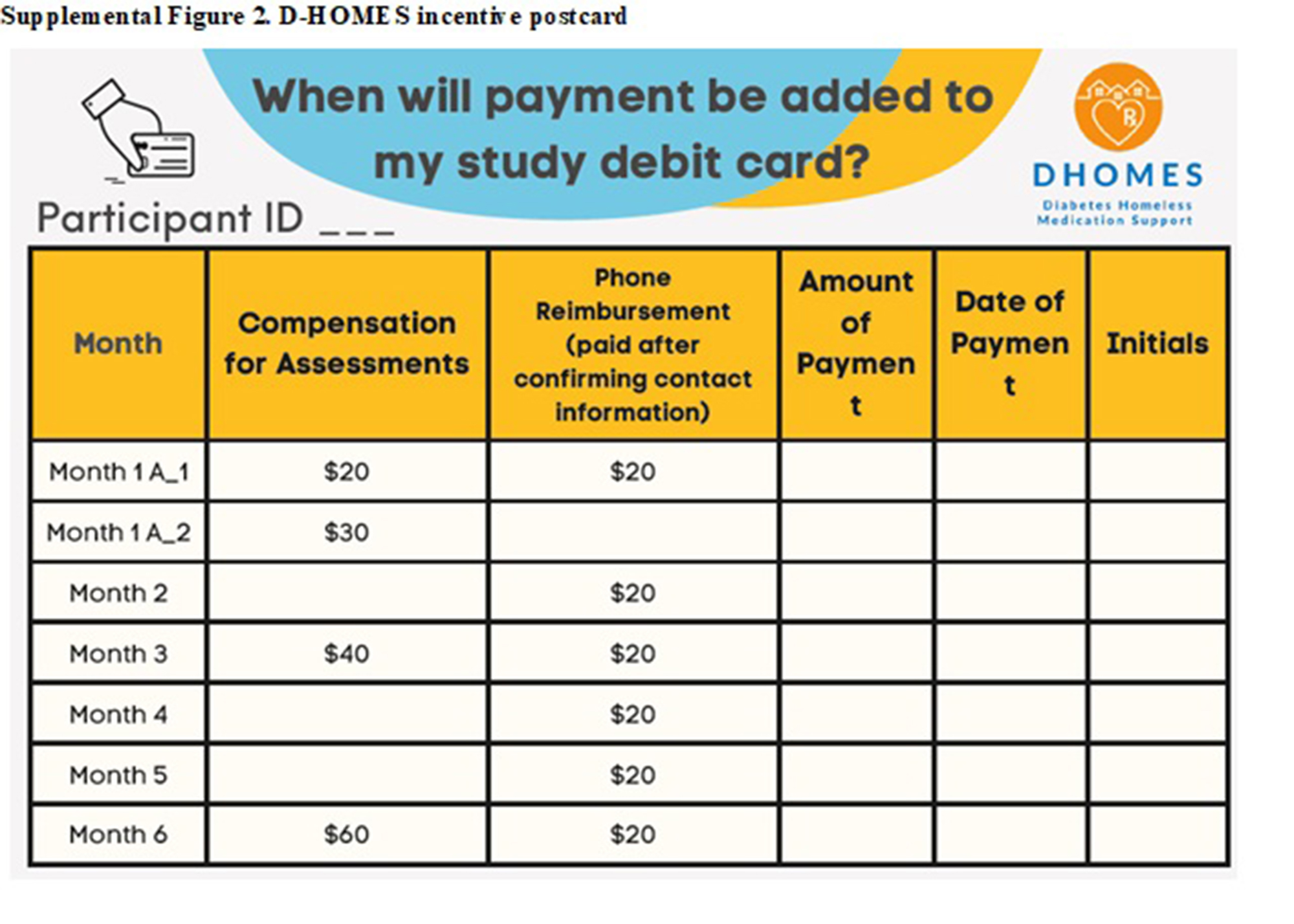

Supplement: Supplementary file 5 [file Image_2.JPEG]

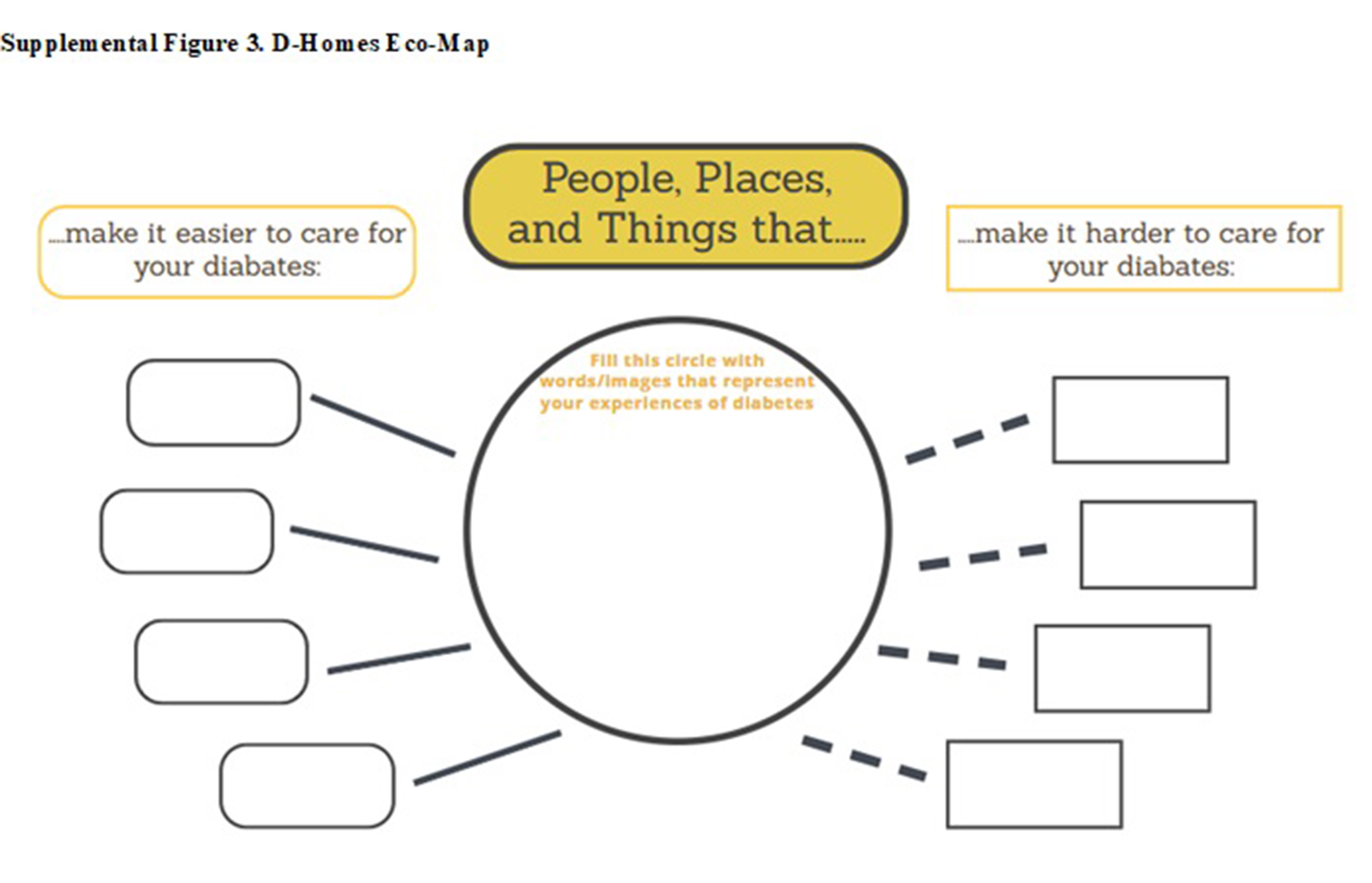

Supplement: Supplementary file 6 [file Image_3.JPEG]
